# High Sensitivity and Wide Range Refractive Index Sensor Based on Surface Plasmon Resonance Photonic Crystal Fiber

**DOI:** 10.3390/s23146617

**Published:** 2023-07-23

**Authors:** Fengmin Wang, Yong Wei, Yanhong Han

**Affiliations:** 1 Liren College, Yanshan University, Qinhuangdao 066004, China; wp921@ysu.edu.cn (Y.W.); hanyanhong@ysu.edu.cn (Y.H.); 2 School of Information Science and Engineering, Yanshan University, Qinhuangdao 066004, China

**Keywords:** photonic crystal fiber, surface plasmon resonance, confinement loss

## Abstract

In order to detect the refractive index (RI) of high refractive index materials such as trichlorobenzene and aniline in the near-infrared and mid-infrared spectra and expand the detection range of the refractive index, a surface plasmon resonance (SPR) photonic crystal fiber (PCF) sensor based on an elliptical sensing channel is proposed for high refractive index detection. The fiber core and the analyte channel are surrounded by two types of air holes with different sizes. When the surface plasmon resonance effect appears at the interface between the fiber core and the elliptical sensing layer, obvious resonance peaks appear in the near-infrared and mid-infrared bands. The full vector finite element method (FEM) is used to study the sensing characteristics of the sensor and the influence of structural parameters on the resonance peak. The results demonstrate that the sensor achieves detection in the refractive index range of 1.41–1.58, in the wavelength range of 1600–3200 nm. The average wavelength sensitivity is 9217.22 nm/RIU, and the refractive index resolution is 10.85 × 10^−6^ RIU. The proposed sensor realizes high refractive index detection in the near-infrared and mid-infrared bands, and obtains an ultra-wide detection range and higher sensitivity. The sensor has broad application prospects in chemical detection, biomedical sensing and other fields, and provides a theoretical reference for the design of a photonic crystal fiber surface plasmon resonance sensor.

## 1. Introduction

Due to the flexible structure design, infinite single-mode transmission, anomalous dispersion, high birefringence, large mode field area, nonlinear effect and many other excellent characteristics [1,2,3,4], photonic crystal fiber (PCF) [5] has incomparable advantages over traditional fiber in many fields. In recent years, PCF has been widely used in the sensing fields. Surface plasmon resonance (SPR) [6,7] has received widespread attention due to its advantages of high sensitivity, unlabeled monitoring and rapid real-time detection. Since SPR is extremely sensitive to small changes in the refractive index, the SPR sensing technology is widely used to measure and detect physical quantities, such as material concentration, temperature and related parameters that may lead to refractive index changes. Therefore, it has broad application prospects in biomedicine, environmental pollution, food safety and the petrochemical industry [8,9,10,11,12]. Optical fiber sensing technology, which combines PCF and SPR, is an emerging and important technology in the sensing field. Photonic crystal fiber based on surface plasma resonance (PCF-SPR) sensors have received extensive attention and applications because of its small probe size, compact structure, remote sensing and other advantages [13].

In 2006, a PCF-SPR sensor was first proposed by Hassani and Skrobogatiy, which attracted widespread attention [14]. Thus, numerous studies [13] have been conducted on PCF-SPR sensors. Various PCF-SPR sensors [13] have been designed and widely used for sensing and detection. In 2020, Tianshu Li et al. [15] proposed an H-type PCF-SPR RI sensor with Ag-graphene layers externally deposited, and the detection range is 1.33–1.41, operating in the wavelength range of 600–1050 nm. Md. Biplob Hossain et al. [16] designed a PCF-based SPR sensor with a quasi D-type dual core structure. It can operate in the 1080–1560 nm wavelength range with an RI detection range of 1.42–1.46. A novel PCF-SPR sensor with three layers of regular hexagonal air holes proposed by Zipeng Guo et al. [5] obtained a wide RI detection range of 1.35–1.46 at 1200–1450 nm. In 2022, Satyendra Jain et al. [17] studied a PCF sensor with an outer surface coated with gold film, and the detection range of the RI is from 1.35 to 1.40 at a wavelength range of 500–1350 nm. In the same year, Ahmed A. Saleh Falah et al. [18] reported a D-shaped PCF sensor with an open microchannel. Its RI detection range is 1.330–1.435, and the operating wavelength is 550–1300 nm. From the above literature, we can see that the detection range of existing reported PCF-SPR RI sensors is generally narrow, and the upper detection limit is relatively low, usually lower than 1.50, and unable to achieve high refractive index detection. However, some solutions have relatively high RI, such as benzene, nitrobenzene and phenylamine. Thus, it is necessary to increase the upper detection limit of RI. In addition, the working band is mainly in the visible light or communication band. Near-infrared sensors can avoid light damage and phototoxicity to biological materials [19,20]. In the mid-infrared spectrum, there is a strong interaction between light and matter at some wavelengths, which makes it possible to detect with high selectivity and sensitivity [21,22]. At the same time, the penetration depth of an evanescent wave is proportional to the working wavelength. Compared with visible light, an evanescent wave in near-infrared and mid-infrared bands has a deeper penetration depth. Therefore, the demand for sensors with a ultra-wide refractive index detection range and ultra-high refractive index detection upper limit working in the near-infrared and mid-infrared bands has increased significantly.

In this paper, a novel PCF-SPR RI sensor is proposed, which can detect liquid samples with a relatively large effective RI in the near-infrared and mid-infrared bands. On the inner side of the large elliptical pore above the fiber core, a nanoscale gold film is coated to stimulate SPR. The liquid analyte is injected into the large elliptical air hole coated with gold film. The FEM [23]-based commercial COMSOL software is used to analyze the sensing characteristics of the fiber and the effects of structural parameters on the resonance wavelength and confinement loss are discussed. The optical fiber sensor can detect the RI of liquid analytes from 1.41 to 1.58 in the 1600–3200 nm band. The average wavelength sensitivity is 9217.22 nm/RIU.

## 2. Structure Design and Theoretical Modeling

**Figure 1:**
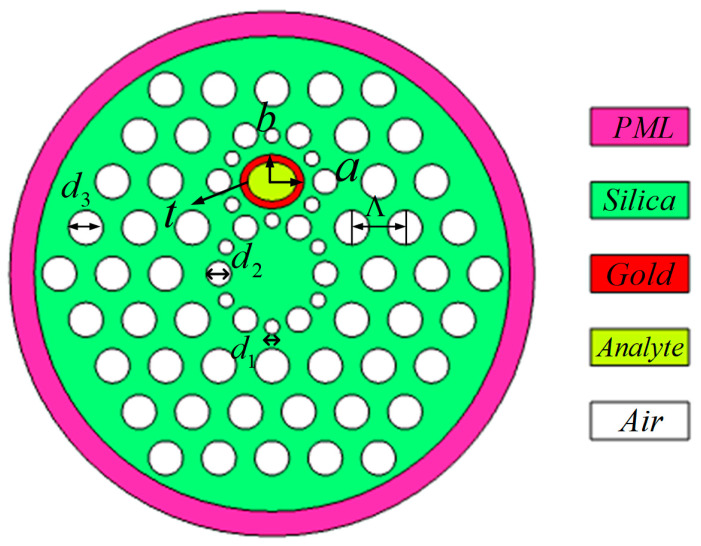
The cross-sections of the proposed SPR-PCF refractive index sensors.

The cross-section of the RI sensor is shown in Figure 1. It includes four layers of pores arranged in a hexagonal manner, the lattice constant is Ʌ = 2170 nm. The fiber core is surrounded by twelve air holes with different sizes in a regular hexagonal way, in which the diameter of the large holes is d_2_ = 800 nm, and the diameter of the small holes is d_1_ = 400 nm. Above the fiber core, there is a hexagon, which is also composed of twelve air holes, whose diameter is the same as that surrounding the fiber core. At the center of the upper hexagon, an elliptical channel serves as the sensing channel of the analyte with its long half axis a = 1500 nm and short half axis b = 1300 nm. The refractive index of analyte is n_a_. The thickness of the gold layer is t = 40 nm. The diameter of other air holes is d_3_ = 1200 nm.

With the development of optical fiber manufacturing technology, the stack-and-draw method, 3D printing method, sol–gel method and other technologies [24,25,26,27] have reached a high level. Among them, the sol–gel method can produce any kind of PCF structure. The method of coating metal film has also been more common [28,29]. Vapor deposition method [28] is a commonly used technology in the process of metal film coating, the high-pressure microfluidic chemical deposition technology [29] uses high pressure gas flow to introduce the precursor of the target material into the air hole, which can achieve uniform, dense and annular sediments in the PCF air holes. Because the optical fiber structure designed in this paper contains elliptical air holes, the manufacturing process is relatively difficult compared with the circular air hole optical fiber, so the optical fiber and its air hole structure can be fabricated by sol–gel method. The gold layer can be deposited on the inner surface of the elliptical pores by high-pressure microfluidic chemical deposition technology. The high aspect ratio of the elliptical air hole leads to the uneven coating, which causes the FWHM broadening. In order to improve the uniformity of the coating, during the fiber drawing process, the smoothness of the microchannel surface should be improved as much as possible, which can be achieved by reducing the furnace temperature to a certain level. In addition, we should strictly control the deposition time, solution flow rate and temperature. During the coating process, first, industrial silica gel is used to selectively block the uncoated holes. Then, the optical fiber is put into the coating equipment.

**Figure 2:**
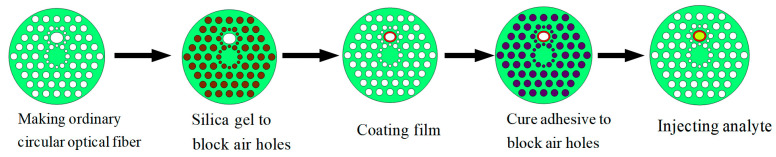
Schematic for fiber fabrication process. The white circle represents the air hole, the brown circle represents the silica gel, and the dark blue circle represents the cure adhesive. The gold layer is marked in red and the analyte is marked in yellow.

The liquid analyte can be filled into the elliptical air hole by capillary phenomenon. First, pores that do not need to be filled should be blocked with curing adhesive, then, the filling of the analyte is realized by capillary absorption. Direct injection method can also be used to fill the liquid analyte into the elliptical air hole. For viscous liquid, in order to avoid the capillary effect and the influence of viscosity on liquid filling, a syringe can be used to pump the liquid into the air hole of the optical fiber through pressure. In order to avoid contaminating the analyte, the sensor should be repeatedly cleaned with alcohol before and after each test to ensure the cleanness of the sensing area. The whole preparation process of optical fiber is shown in Figure 2.

PCF-SPR RI sensors can be divided into two categories based on the location of metal materials: internal metal coating [30,31] and external metal coating [9,11]. The optical fiber designed in this paper belongs to the first type. Metal materials are coated in certain air holes inside the PCF. When the analyte is filled into the air holes coated with metal film, the characteristics of the analyte can be measured by analyzing the spectral changes in transmitted light. The production process of this type of optical fiber is cumbersome and costly. However, because the metal layer is located in the air hole, it can be free from the oxidation of the external environment, and the sensor has high sensitivity and stable performance. Unlike the first type of PCF-SPR RI sensor, the metal material is coated on the outer surface of the PCF, such as D [9] and multicore PCF sensors [32]. The manufacturing process is relatively simple, but the metal plasmon material is easily oxidized, which affects the reliability of the sensor.

**Figure 3:**
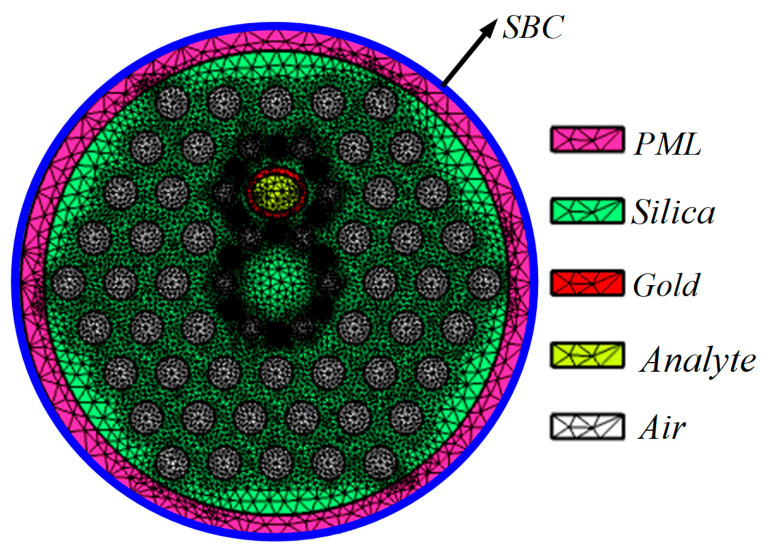
FEM meshing and boundary condition setting.

In the simulation, we use the FEM to study the mode characteristics and the dispersion curves of the fundamental mode and the surface plasmon polaritons (SPP) mode. A perfectly matched layer (PML) is added to the periphery of the cladding to absorb reflection. The boundary condition of the periphery is the scattering boundary condition (SBC). Its physical meaning is that it is completely transparent to some specific known wave vectors. In this paper, the free triangle mesh is used to discretize the whole solution domain. The maximum cell size is 2140 nm, and the minimum cell size is 42.8 nm. The whole calculation region contains 19,326 solution elements, with a total degree of freedom of 135,387. The meshing results are shown in Figure 3.

The RI of air is set as 1. The background material of PCF is SiO_2_ and its material dispersion can be obtained from the Sellmeier equation [32]. We can obtain the dielectric constant of gold through the Drude–Lorentz model [32]. In addition, the confinement loss of the mode can be obtained from the following formula [33]:

(1) α=8.686×2πλ×Im(neff)×107dB/cm

where λ represents the wavelength of the incident light, the unit is nanometer and Im(neff) represents the imaginary part of the effective RI.

**Figure 4:**
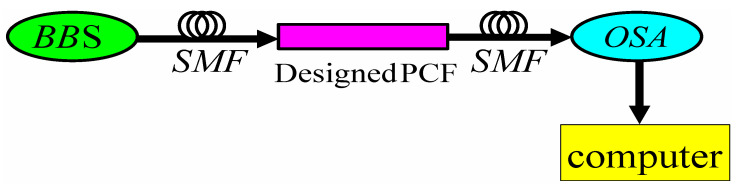
Schematic of the proposed photonic crystal fiber sensor set-up.

The PCF-SPR RI sensor designed in this paper can adopt the system structure as shown in Figure 4, including broadband light source (BBS), single-mode fiber (SMF), PCF-SPR RI sensor, optical spectrum analyzer (OSA) and computer. The experimental process can be carried out in the following steps. First, the liquid analyte is sucked into the elliptical channel by means of “siphon”, and then the BBS is used to generate broadband continuous beam. The incident light is transmitted to the PCF-SPR through SMF. The loss information is transmitted to the OSA through an SMF. Finally, the results are calculated and analyzed by computer.

**Figure 5:**
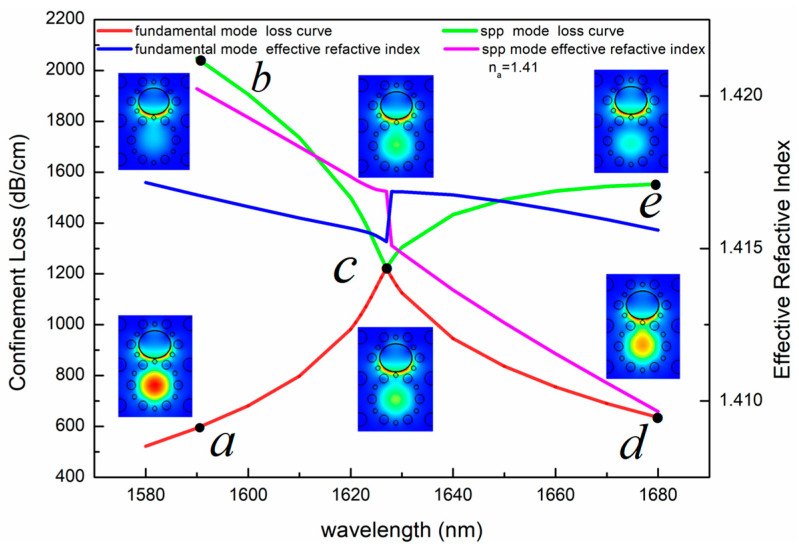
Calculated loss spectrum and dispersion relations of the fundamental mode and the SPP mode at n_a_ = 1.41. Insets are electric field distributions of various wavelength. At points a and b, the corresponding wavelength is 1590 nm, at point c, the corresponding wavelength is 1627 nm, and at points e and d, the corresponding wavelength is 1680 nm.

The dispersion relations and loss curves of the fundamental mode and the SPP mode with n_a_ = 1.41 are calculated as shown in Figure 5. The insets in Figure 5 correspond to electric field distributions of the two modes at wavelengths of 1590 nm, 1627 nm and 1680 nm, respectively. Figure 5 illustrates that the fundamental mode and the SPP mode produce a resonance at point c with a wavelength of 1627 nm. At point c, the confinement losses of the two modes are equal, and the effective RI difference is the smallest. In this case, the coupling of the two modes meets the conditions of phase matching and loss matching. The corresponding mode field conversion occurs between the two modes, and the anti-crossing effect takes place. Thus, point c is called the avoided crossing point. This is known as complete coupling.

## 3. Simulation Results and Discussions

### 3.1. Performance Analysis

The basic principle of the SPR sensor is that the evanescent wave generated by incident light at the interface between the optical fiber and the metal layer causes the coherent oscillation of free electrons, thus generating the surface plasma wave. When the evanescent wave resonates with the SPP, the energy is transferred from the photon to the surface plasma. In this way, the surface plasma wave can absorb most of the energy of the emitted light, resulting in a loss peak of the reflected light intensity. The effective RI of SPP mode is affected by the RI of the analytes. The change in the RI of the analytes changes the resonance wavelength of the SPP mode and the fundamental mode, and the different RI of the analyte corresponds to a different resonance wavelength. Thus, the RI of the analyte is detected by measuring the resonant wavelength.

**Figure 6:**
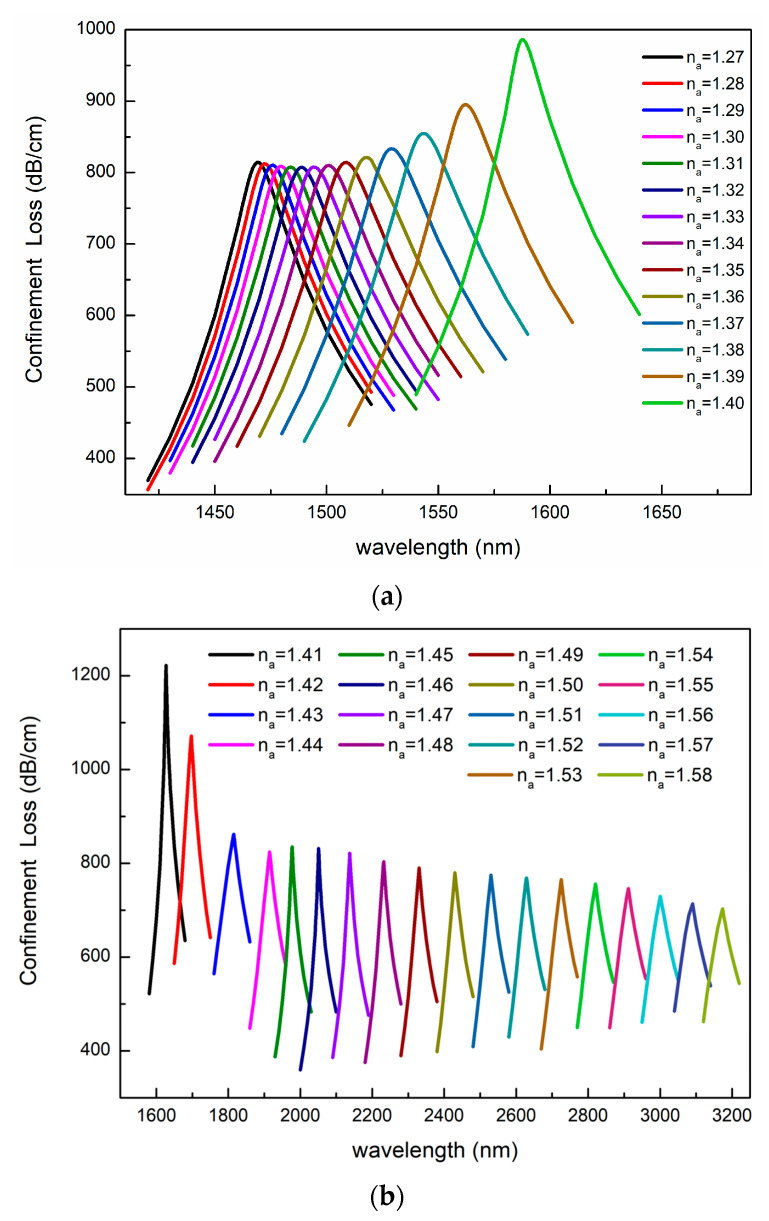
Variation in confinement losses with wavelength for different analytes in the RI ranges of (**a**) 1.27–1.40 and (**b**) 1.41–1.58.

Firstly, we studied the optical fiber sensing characteristics under the different Ris of liquid analytes. In the simulation, we varied the RI of the liquid analyte and the structural parameters (Ʌ = 2170 nm, d_1_ = 400 nm, d_2_ = 800 nm, d_3_ = 1200 nm, a = 1500 nm, b = 1300 nm, t = 40 nm) remained unchanged. Figure 6 depicts the relationship between the confinement loss and wavelength as the RI changes. It can be seen intuitively that the resonance wavelength is red shifted with the increasing RI of the liquid analyte. This is because the RI of the SPP mode increases as the RI of the liquid analyte increases, while that of the fundamental mode changes little, which causes the resonance point to shift toward longer wavelengths. At the same time, the numerical value of the confinement loss decreases as the RI of the analyte increases.

**Figure 7:**
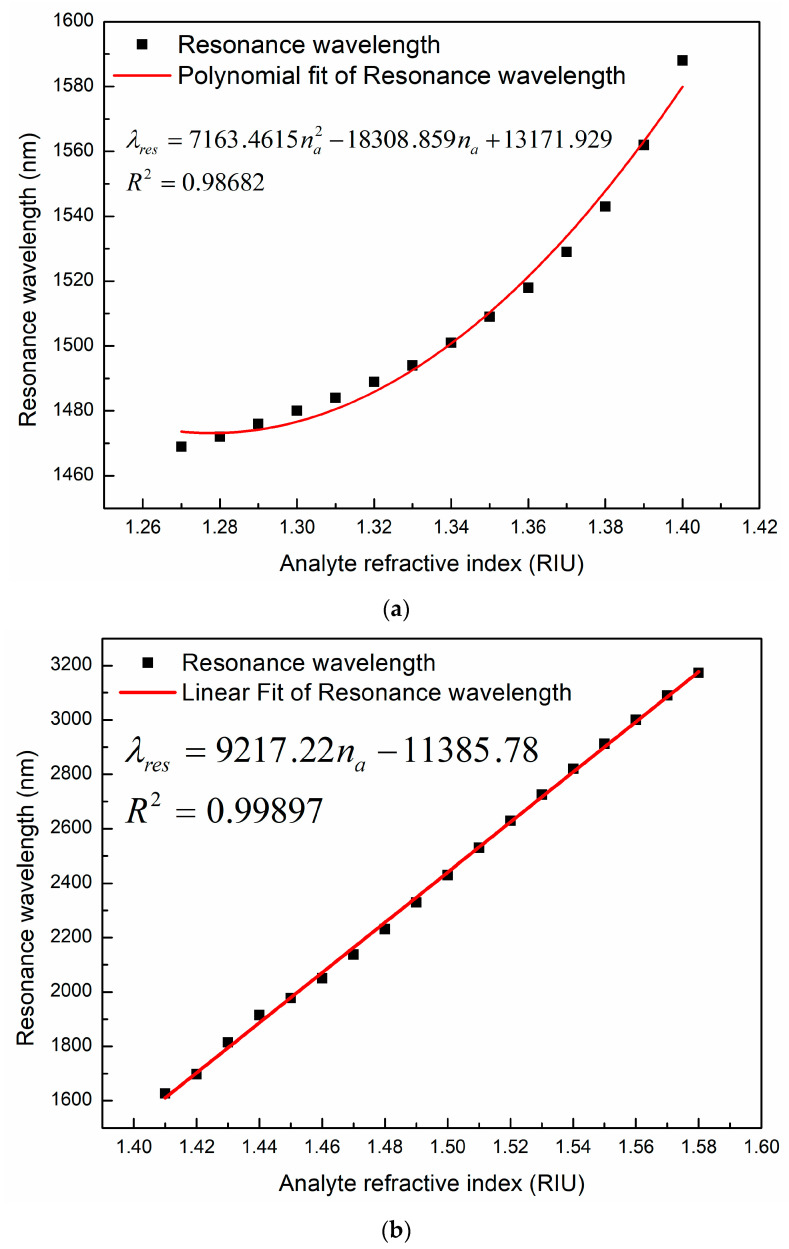
Confinement loss spectra as the analyte RI is changed from (**a**) 1.27 to 1.40 and (**b**) 1.41 to 1.58.

In order to achieve the relationship between the resonant wavelength and the RI of the analyte, we used Origin software to fit them. The fitting results are shown in Figure 7. The fitting equations and the adjusted R-square (ARS) values are shown below.

(2) $$\lambda_{res}=7163.4615n_{a}^{2}-18308.859n_{a}+13171.929$$

$$1.27\leq n_{a}\leq 1.40\;\;\;ARS=0.98682$$

(3) $$\lambda_{res}=9217.22n_{a}-11385.78,\;1.41\leq n_{a}\leq 1.58,\;ARS=0.99897$$

where $\lambda_{res}$ represents the resonant wavelength, and $n_{a}$ is the RI of the analyte. According to the fitting results, when the RI of the analyte changes from 1.27 to 1.40, the relationship between the resonant wavelength and the RI meets the quadratic curve as shown in Figure 7a. When the RI changes from 1.41 to 1.58, the resonant wavelength and RI satisfy the linear relationship as shown in Figure 7b.

Wavelength sensitivity is an important parameter to measure the sensor performance. We can evaluate the sensing performance of the PCF-SPR sensor by calculating the wavelength sensitivity. The wavelength sensitivity is often defined as [34]:

(4) $$S_{\lambda }=\partial \lambda_{peak}/\partial n_{a}\left[\mathrm{nm}/\mathrm{RIU}\right]$$

where $\partial \lambda_{peak}$ refers to the shift distance of the resonance peak and $\partial n_{a}$ is the variation in the RI of the analyte. Therefore, the slope of the fitting curve represents the wavelength sensitivity and the slope of the linear fitting line represents the average wavelength sensitivity. The fitting results show that there is a good linear relationship in the range of RI 1.41–1.58. Since the resonant wavelength and RI do not meet the linear relationship and the wavelength sensitivity is relatively smaller in the RI range of 1.27–1.40, we choose 1.41–1.58 as the RI detection range of the sensor. According to the fitting results, the average wavelength sensitivity of our proposed sensor is 9217.22 nm/RIU when n_a_ varies from 1.41 to 158. Due to the high sensitivity and linearity, the sensor we propose has considerable advantages in the measurement of high RI analytes.

RI resolution is another parameter to evaluate the performance of the sensor, which represents the minimum change in the refractive index of the sample that the sensor can detect. It is defined as the equation [35]:

(5) $$R=\partial n_{a}\times \partial \lambda_{\min}/\partial \lambda_{peak}\left[\mathrm{RIU}\right]$$

where $\partial \lambda_{\min}$ indicates the wavelength resolution of the instrument. Assuming the optimal wavelength resolution of the spectrometer (such as Yokogawa AQ6376 fiber spectrometer) used is 0.1 nm, the RI resolution of the proposed sensor can reach 10.85 × 10^−6^ RIU.

**Figure 8:**
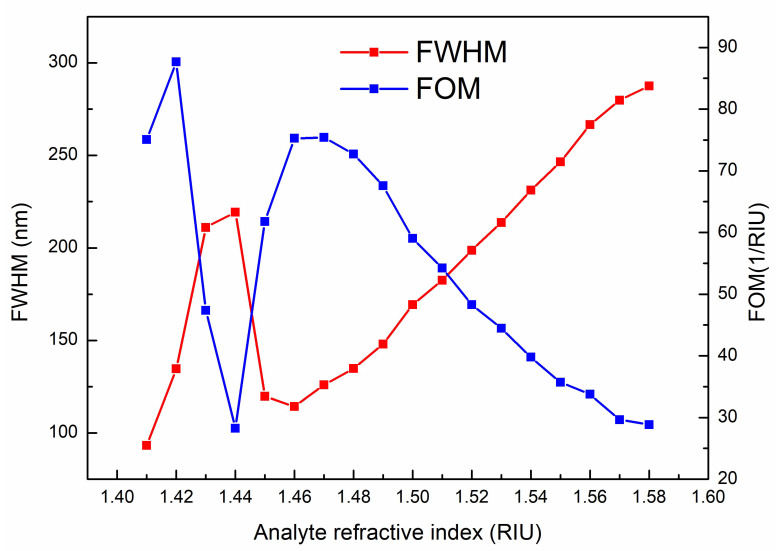
Relationship between FOM, FWHM and RI of analytes.

**Figure 9:**
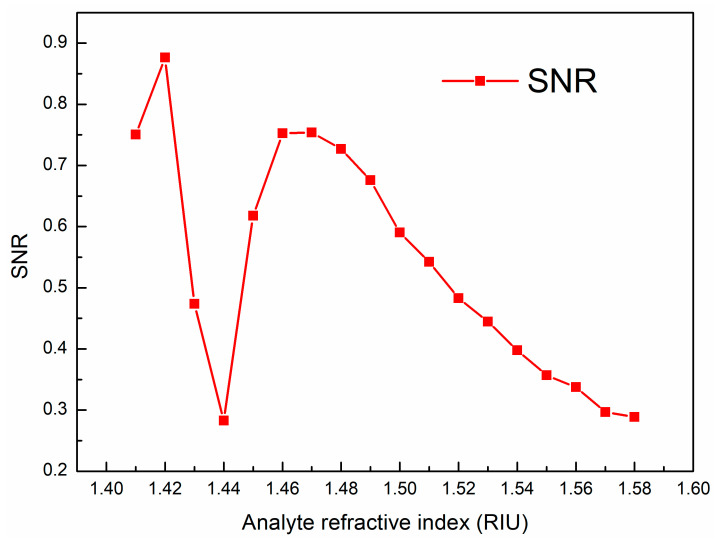
Relationship between SNR and RI of analytes.

In addition to wavelength sensitivity and RI resolution, full-width at half-maximum (FWHM), the figure of merit (FOM) and signal-to-noise ratio (SNR) are also important parameters for evaluating the performance of the sensor. FWHM refers to the peak width at half the height of the loss spectrum peak. For the PCF-SPR sensor, the greater the sensitivity, the better the performance. However, if the FWHM of the resonant peak is too large, the resolution will be reduced and the resonant peak tracking will be difficult. Therefore, in order to fully understand the performance of the SPR sensor, we introduce the FOM. FOM is a physical quantity to evaluate the overall performance of sensor devices, which can be obtained from the following formula [36].

(6) $$\mathrm{FOM}=\frac{S_{\lambda }}{\mathrm{FWHM}}$$

where $S_{\lambda }$ is the wavelength sensitivity corresponding to the RI of the analyte. SNR is another parameter affecting sensor performance, which can be obtained from the following expression [35].

(7) $$\mathrm{SNR}=\frac{\Delta \lambda_{R}}{\mathrm{FWHM}}$$

where $\Delta \lambda_{R}$ represents the difference between two adjacent resonant wavelengths. Figure 8 describes the relationship between the FOM, FWHM and analyte RI. The FOM reaches the maximum value of 87.648 RIU^−1^ when the RI of the analyte is 1.42, and then decreases. Next, the FOM reaches its second largest peak value when the RI of the analyte is 1.47, and then begins to slowly decrease. The change trend of the FWHM is basically opposite to that of the FOM. The sensor’s SNR is shown in Figure 9. The variation trend of the SNR with RI is basically consistent with that of the FOM. The highest SNR value is found to be 0.876 when the RI of the analyte is 1.42.

Table 1 compares the performance of the proposed PCF-SPR sensor and other optical fiber sensors based on SPR in three aspects: RI detection range, working wavelength and average wavelength sensitivity. We find that compared with other sensors, our proposed sensor has a much wider RI detection range. At the same time, the optical fiber sensor we designed has a very high upper detection limit of the analyte RI, which makes it possible to measure some high RI organic chemical liquid samples. Furthermore, compared with these sensors, the proposed sensor can operate in an ultrabroad wavelength range of 1600–3200 nm, which makes the sensor applicable for detection in the near-infrared and mid-infrared bands. Thus, among the many PCF-SPR sensors, the sensor proposed in this work has significant advantages and competitiveness in the application of RI detection.

### 3.2. Effect of Different Structural Parameters on the Fiber Sensor

**Figure 10:**
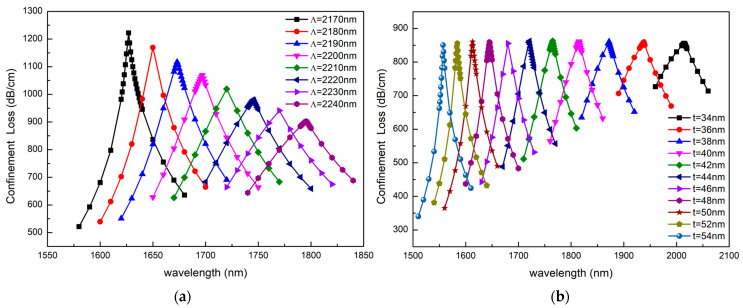
(**a**) The confinement loss depending on the wavelength as Ʌ increases from 2170 nm to 2240 nm. The other structural parameters are n_a_ = 1.41, d_1_ = 400 nm, d_2_ = 800 nm, d_3_ = 1200 nm, a = 1500 nm, b = 1300 nm, t = 40 nm. (**b**) The confinement loss depending on the wavelength as t increases from 34 nm to 54 nm. The other structural parameters are n_a_ = 1.43, d_1_ = 400 nm, d_2_ = 800 nm, d_3_ = 1200 nm, a = 1500 nm, b = 1300 nm, Ʌ = 2170 nm.

**Figure 11:**
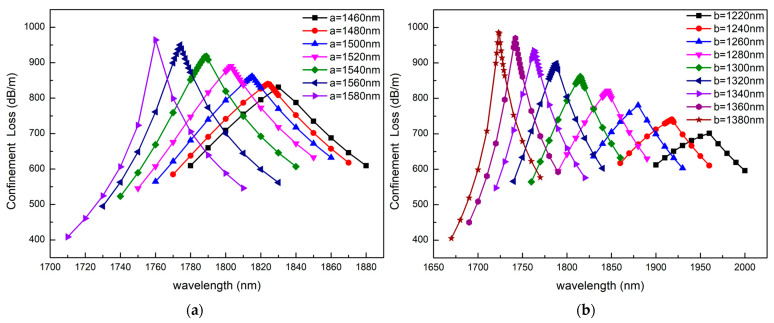
The confinement loss depending on the wavelength as (**a**) long half axis increases from 1460 nm to 1580 nm and (**b**) short half axis increases from 1220 nm to 1380 nm. The other structural parameters are n_a_ = 1.43, d_1_ = 400 nm, d_2_ = 800 nm, d_3_ = 1200 nm, Ʌ = 2170 nm, t = 40 nm.

**Figure 12:**
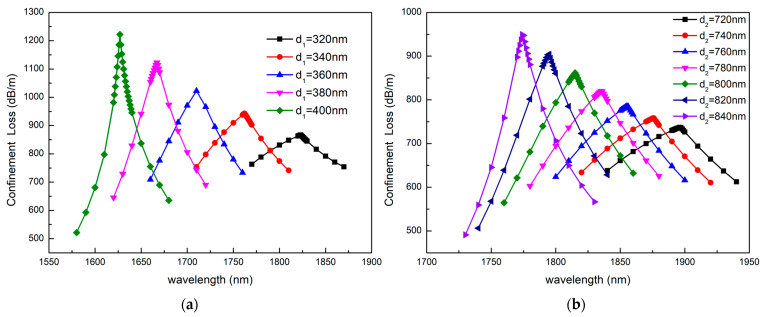
The confinement loss depending on the wavelength as (**a**) d_1_ increases from 320 nm to 400 nm and (**b**) d_2_ increases from 720 nm to 840 nm. The other structural parameters are n_a_ = 1.41, d_3_ = 1200 nm, Ʌ = 2170 nm, a = 1500 nm b = 1300 nm, t = 40 nm.

In order to investigate the impact of fiber structural parameters on sensing performance, we discussed the changes in loss peaks when structural parameters change. The results are shown in Figure 10, Figure 11 and Figure 12. As shown in Figure 10a, we make the lattice constant Ʌ vary from 2170 nm to 2240 nm while keeping all other structural parameters unchanged. It can be established that the confinement loss of the fundamental mode decreases when the lattice constant Ʌ is increasing. This is because the lattice constant represents the lattice period of the photonic crystal, and with the increase in the lattice constant, the resonance intensity between the fundamental mode and the SPP mode decreases. Thus, the energy is limited to the core and the confinement loss reduces. Meanwhile, the resonance wavelength is red shifted with the increase in the lattice constant Ʌ.

Figure 10b illustrates the effect of gold layer thickness on sensing characteristics. It can be seen from Figure 10b that the surface plasma wave is very sensitive to the thickness of the gold layer. With the increase in the gold layer from 34 nm to 54 nm, the resonance peak shifts from 2015 nm to 1557 nm and the FWHM becomes narrower; however, the height of the loss peak at the resonant wavelength hardly changes. The above simulation results can be explained as follows. As the thickness of the gold layer increases, the number of carriers in the metal increases, the number of electrons that can participate in surface plasma resonance increases and the energy increases, so the resonance wavelength moves to a shorter wavelength and a blue shift occurs. At the same time, with the thickness of the gold layer increasing, the phase delay effect occurs when the penetration depth of the electric field is smaller than the thickness of the gold layer. When the phase delay effect plays a dominant role, the resonance intensity and the loss peak decreases. When the influence mechanism of increasing the number of free electrons plays a dominant role, the resonance intensity and the loss peak increases. When these two mechanisms are equalized, the resonance intensity and the loss peak remain unchanged. Therefore, we can flexibly adjust the sensor performance by changing the thickness of the gold layer.

Figure 11 shows that the size of the ellipse has a significant effect on the resonance wavelength and the resonance peak intensity. Figure 11a depicts the confinement loss as the long half axis increases from 1460 nm to 1580 nm, and Figure 11b depicts the confinement loss as the short half axis increases from 1220 nm to 1380 nm. According to Figure 11a,b, with the long half axis and short half axis increasing, the resonance wavelength is blue shifted and the confinement loss of the fundamental mode increases as a whole. This is because the larger the size of the ellipse, the smaller the distance between the fiber core and the plasmonic material. Therefore, the coupling strength between the fundamental mode and the SPP mode is affected by the size of the ellipse. With the increase in the ellipse size, the distance between the gold layer and the fiber core becomes shorter, which leads to stronger resonance and a higher loss peak.

Figure 12 describes the loss curve of the sensor when the air holes around the fiber core and sensing channel changes. Figure 12a describes the confinement loss as d_1_ increases from 320 nm to 400 nm, and Figure 12b describes the confinement loss as d_2_ increases from 720 nm to 840 nm. As can be seen from Figure 12a,b, the resonance wavelength undergoes a blue shift and the resonance peak increases with the diameters of the two types of air holes increasing. This is because the increase in pore size promotes the leakage of the mode field from the core, resulting in worse constraints on the core. Thus, by changing the size of the two types of air holes, we can adjust the resonance wavelengths of the two modes.

## 4. Conclusions

A novel PCF-SPR RI sensor is presented in this paper. Pores of different sizes surround the core and analyte channel in a hexagonal manner. In this structure, SPR is excited by coating a gold film inside the elliptical channel. The theoretical model was analyzed using the full vector FEM, and the influence of the fiber structure parameters on its sensing characteristics was studied. The results show that the optical fiber SPR sensor can obtain the RI detection range of 1.41~1.58 in a wide band of 1600–3200 nm. The average wavelength sensitivity and RI resolution can reach 9217.22 nm/RIU and 10.85 × 10^−6^ RIU, respectively. The proposed sensor not only achieves an ultra-high upper limit of RI detection and an ultra-wide RI detection range, but also realizes near-infrared and mid-infrared sensing and a higher average wavelength sensitivity. Therefore, we can widely apply this sensor to high-precision detection of high RI substances in the fields of biomedicine and chemistry.

## Figures and Tables

**Table 1:** Performance comparison with the previously reported PCF-SPR sensors.

References	Detecting Range of RI (RIU)	Operation Wavelength Range (nm)	Average Sensitivity (nm/RIU)
[37]	1.33–1.43	1000–1480	2150
[38]	1.35–1.40	600–900	3330
[39]	1.355–1.385	1300–1600	5200
[40]	1.36–1.41	600–2000	15,428.5
This work	1.41–1.58	1600–3200	9217.22

## Data Availability

Data sharing is not applicable to this article.
